# Altered functional connectivity and network excitability in a model of cortical dysplasia

**DOI:** 10.1038/s41598-023-38717-2

**Published:** 2023-07-30

**Authors:** A. Aquiles, T. Fiordelisio, H. Luna-Munguia, L. Concha

**Affiliations:** 1 Institute of Neurobiology, Universidad Nacional Autónoma de México, Campus Juriquilla, Querétaro, Querétaro Mexico; 2 Facultad de Ciencias, Universidad Nacional Autónoma de México, Mexico City, Mexico; 3 Laboratorio Nacional de Soluciones Biomiméticas para Diagnóstico y Terapia LaNSBioDyT, Universidad Nacional Autónoma de México, Mexico City, Mexico

**Keywords:** Epilepsy, Development of the nervous system, Diseases of the nervous system

## Abstract

Focal cortical dysplasias (FCDs) are malformations of cortical development that often result in medically refractory epilepsy, with a greater incidence in the pediatric population. The relationship between the disturbed cortical morphology and epileptogenic activity of FCDs remains unclear. We used the BCNU (carmustine 1-3-bis-chloroethyl-nitrosourea) animal model of cortical dysplasia to evaluate neuronal and laminar alterations and how these result in altered activity of intracortical networks in early life. We corroborated the previously reported morphological anomalies characteristic of the BCNU model, comprising slightly larger and rounder neurons and abnormal cortical lamination. Next, the neuronal activity of live cortical slices was evaluated through large field-of-view calcium imaging as well as the neuronal response to a stimulus that leads to cortical hyperexcitability (pilocarpine). Examination of the joint activity of neuronal calcium time series allowed us to identify intracortical communication patterns and their response to pilocarpine. The baseline power density distribution of neurons in the cortex of BCNU-treated animals was different from that of control animals, with the former showing no modulation after stimulus. Moreover, the intracortical communication pattern differed between the two groups, with cortexes from BCNU-treated animals displaying decreased inter-layer connectivity as compared to control animals. Our results indicate that the altered anatomical organization of the cortex of BCNU-treated rats translates into altered functional networks that respond abnormally to a hyperexcitable stimulus and highlight the role of network dysfunction in the pathophysiology of cortical dysplasia.

## Introduction

Focal cortical dysplasias (FCDs) are a type of cortical development malformation that constitutes a frequent cause of refractory epilepsy in the pediatric population and in adults^1–5^. Clinically, FCDs show great variability in their morphology, extension, and location. Whereas some are large and easily identifiable with conventional imaging methods, others are subtle and difficult to find, which may preclude the possibility of neurosurgical treatment^2,6^. The mechanisms driving epileptogenesis in FCDs are not fully understood due to the impossibility of performing longitudinal evaluations in patients before the onset of seizures.

Animal models of FCD provide a good opportunity to study the morphological features and pathophysiological mechanisms of epilepsy related to human cortical malformations. The carmustine 1-3-bis-chloroethyl-nitrosourea (BCNU) model has been widely described in rats, inducing in the offspring laminar disarrangement, abnormal cell morphologies, and features similar to those seen in humans with FCD type IIa^7–11^. While animals with this induced lesion do not present spontaneous seizures, some studies have described an augmented excitability of the malformed cortex^12^ and enhanced susceptibility of the lesioned animals to trigger seizures^8,13,14^. In this study, we used the BCNU model to evaluate the physiological repercussions of the morphological alterations seen during early life and determine how these malformations facilitate the generation of abnormal electrical activity. For this, large field-of-view calcium images of live cortical slices allowed us to analyze the spatio-temporal cortical dynamics and how they change after pharmacologically induced stimulation.

Based on the premise that alterations of cellular communication are involved in epilepsy development^15^, the aim of the present study is to understand the link between cortical morphological alterations and intracortical connectivity before, during, and after an external stimulus is applied. BCNU-treated animals showed cortical dislamination, atypical neuronal morphology, and altered baseline connectivity patterns that abnormally responded to the external stimulus.

## Results

### M1 micro-structure characteristics

**Figure 1:**
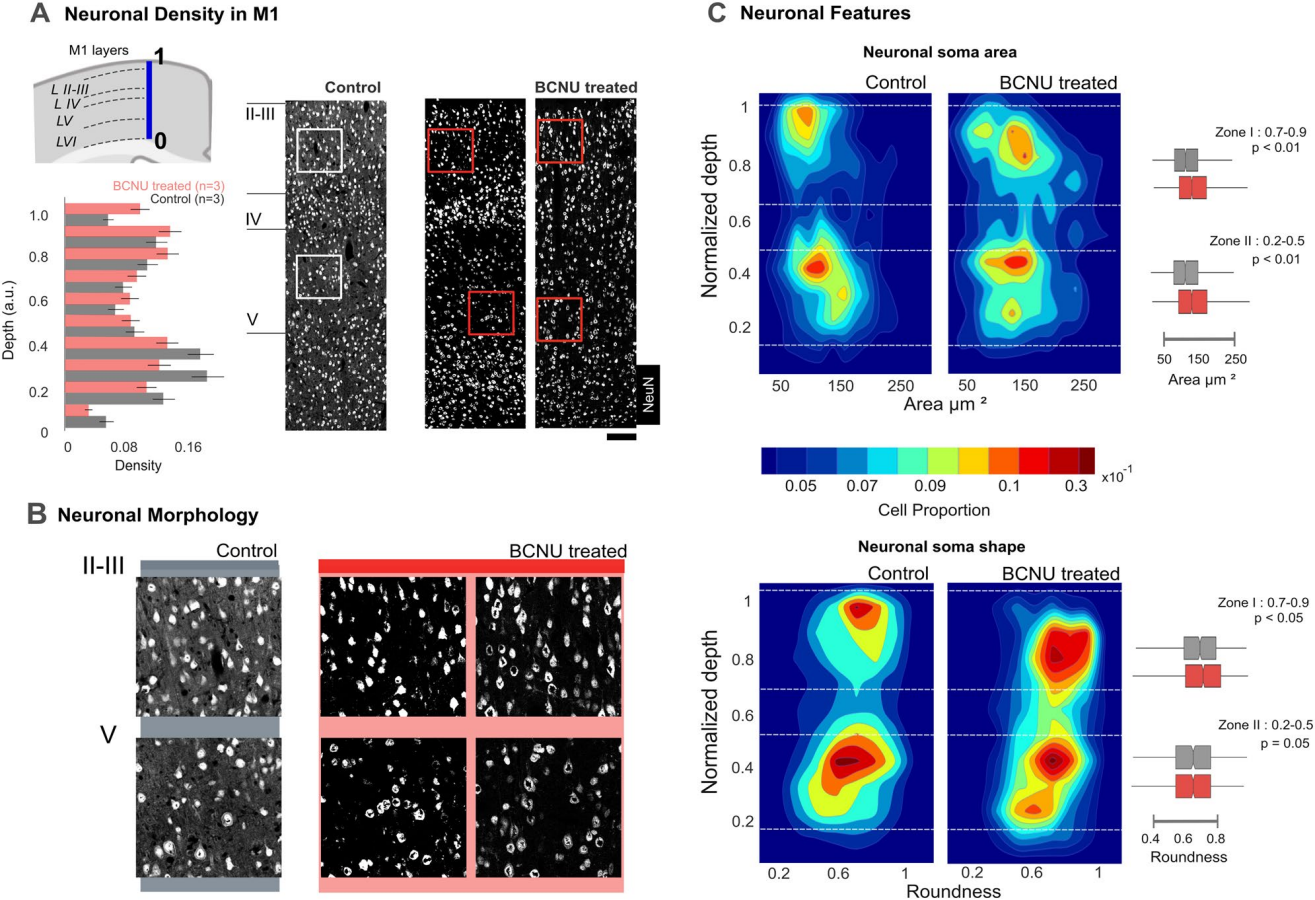
Characterization of microstructure. (**A**) Neuron density along the M1 cortex. Cortical depth was normalized (0: deepest portion to 1: pial boundary), shown as a blue line; dashed lines indicate approximate layer boundaries. NeuN immunolabeling was used to estimate neuronal density and morphology. Spatial profiles of neuronal density are shown (n = 3 animals per group; bars and lines indicate mean and standard error, respectively; light gray—Control, red—BCNU treated). The right panel shows a control and two experimental animals (scale bar: 200 μm). Colored squares are amplified (250 μm) in (**B**). (**C**) Morphological descriptors as a function of depth for the pooled distributions of neurons of all animals, divided by group. Dashed lines delimit Zone I (0.9–0.7 normalized depth), showing enlarged area and roundness; and Zone II (0.5–0.2 normalized depth), showing increased area; box plots of each zone represent the distributions of neuronal area and roundness (p values for between-group Student’s t-tests; Control 605 ± 73, BCNU 687 ± 50 cells). Data from individual animals are illustrated in Supplementary Fig. 1.

**Figure 2:**
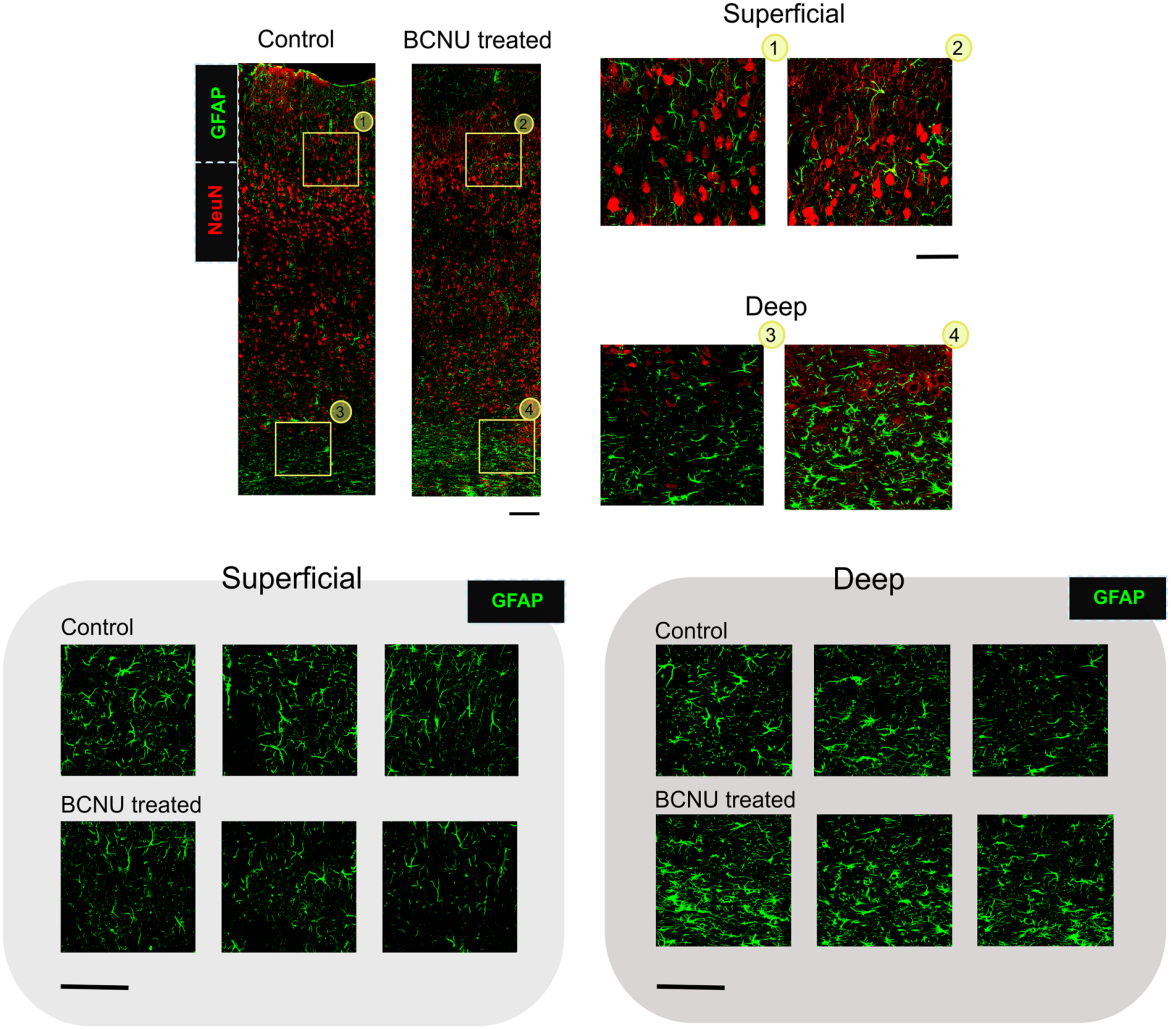
GFAP qualitative evaluation in M1. Top panel shows two representative examples, with immunolabels for NeuN (red), and GFAP (green), in one Control (left) and one BCNU-treated (right) animals; scale bar = 150 μm. The right panels show enlarged views of superficial and deep cortical regions labeled with numbers corresponding to the area and group (Control 1,3; BCNU treated 2,4); scale bar = 50 μm. Bottom panels show three different specimens per group, with magnified views of approximately the same locations as in the top-right panels, immunolabeled for GFAP (green); scale bar = 100 μm.

Cerebral cortex microstructure was evaluated at P30 using a neuron-specific marker (anti-NeuN). The spatial profile of neuronal density as a function of cortical depth was qualitatively similar between groups (Fig. 1A). Neuronal morphology is illustrated in Fig. 1A,B. Qualitative evaluation shows that neurons of BCNU-treated animals are slightly larger than those from control animals, which was confirmed by quantitative evaluation of their area (Fig. 1C, top panel), and subtle yet statistically significant increase of roundness of BCNU-treated animals neurons was seen in the superficial portion of the cortex (Zone 1 in Fig. 1C, bottom panel). These changes coincide with previous reports^7^ and confirm the presence of neuronal alterations in the early stages of their lives. Immunofluorescence of glial fibrillary acidic protein (GFAP) demonstrated qualitatively reduced glial density in the superficial layers and increased branching and widening of astrocytic cell bodies in the deep cortical layers, as compared to controls (Fig. 2).

### Evaluation of cortical layers

**Figure 3:**
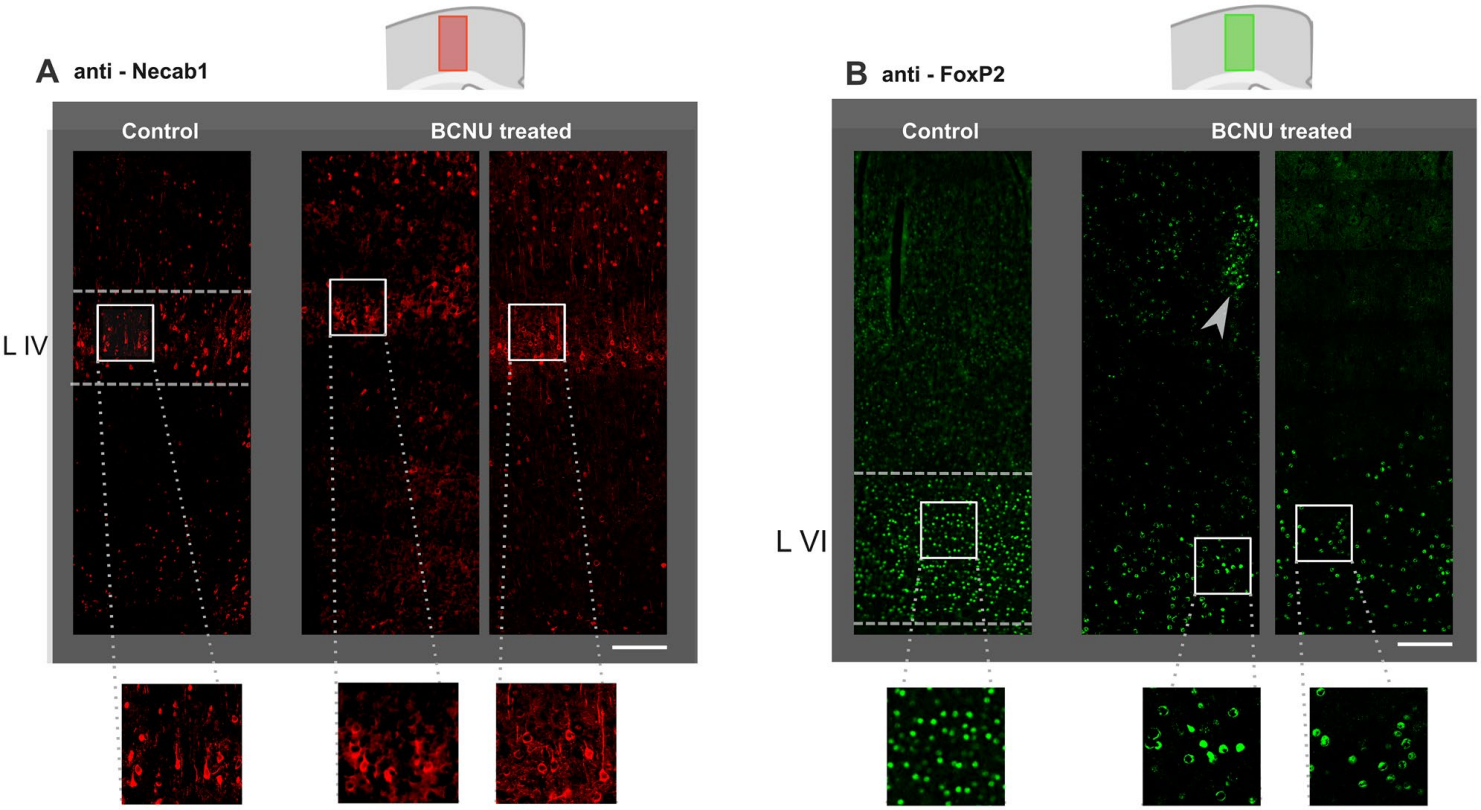
Layer-specific immunofluorescence in M1. (**A**) In control animals, Necab1 clearly delineates layer IV (dashed lines), with little expression in more superficial layers. Two experimental animals show disorganization of layer IV and more expression in layers II to III. (**B**) FoxP2 is normally expressed in layer VI, which shows a sharp upper boundary in control animals (dashed line). Experimental animals show blurring of the layer VI boundary and clusters of heterotopic neurons in superficial layers (gray arrowhead). Scale bar and zoom examples: 200 μm. Both schematic representations of the cortex make reference to the location and extent of the images.

Two immunofluorescent labels were used to identify neurons that normally reside in specific cortical layers to qualitatively show alterations of cortical layering, namely Necab1 (layer IV) and FoxP2 (layer VI). Evaluation at P30 showed that in controls, Necab1-labeled neurons are spatially homogeneous and their presence is limited to layer IV (Fig. 3A). In contrast, layer IV borders in experimental animals are more difficult to ascertain, with a higher number of labeled neurons in layers II-III. Moreover, the spatial boundaries of Necab1-labeled neurons are less specific, and an increased Necab-1 signal in layers II–III can be detected. FoxP2-labeled neurons were clearly limited to layer VI in control animals. On the other hand, BCNU-treated animals showed a poorly delimited layer VI, and clusters of labeled ectopic neurons could be identified in more superficial layers (Fig. 3B).

### Evaluation of calcium time series

**Figure 4:**
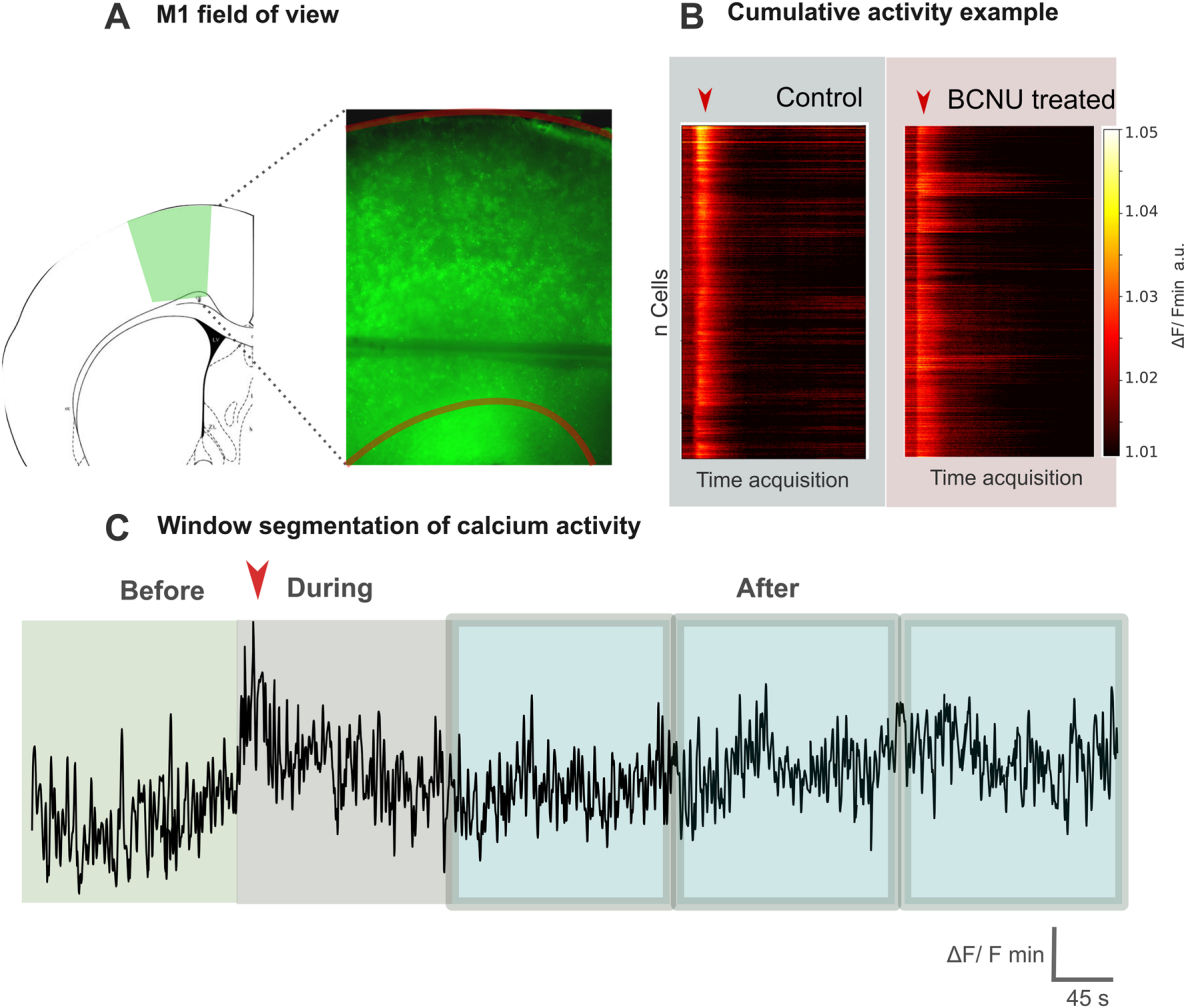
Evaluation of calcium time series. (**A**) Example of the large field-of-view for a [Ca^2+^]_i_ signal recording (encompassing all cortical layers), overlaid on the Paxinos and Watson atlas (2006) for reference. Red lines represent the pial boundary (top) and gray-white matter interface (bottom). (**B**) Population activity of one control and one experimental animal, with each row indicating each neuron’s color-coded signal amplitude (ΔF/Fmin) over time. Arrowheads indicate the pilocarpine stimulus. (**C**) Enlarged view of an example of a [Ca^2+^]_i_ signal for a control animal indicating the selected time windows before, during, and after pilocarpine stimulation (arrowhead) considered for network analyses (see Fig. 6).

**Figure 5:**
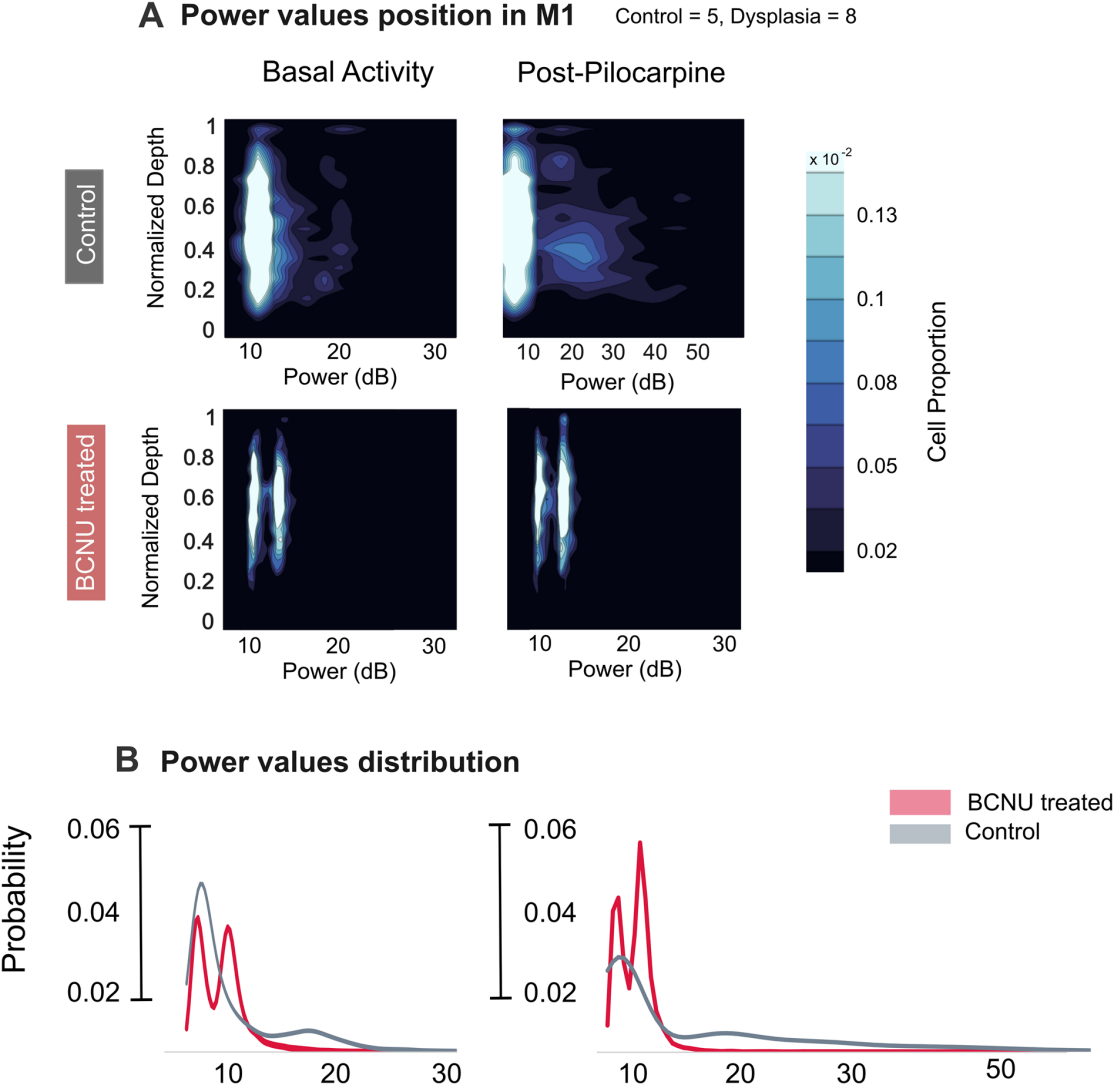
Signal power during basal and post-stimulus activity. (**A**) Cell signal power along the cortical depth during basal activity (left column) and post-pilocarpine (right column); the color scale represents the cell proportion of the total number of neurons evaluated. (**B**) One-dimensional power distributions (i.e., similar to (**A**) but across all cortical depths) during basal activity (left) and post-pilocarpine (right). A time-dependent analysis is shown in Supplementary Fig. 2.

**Figure 6:**
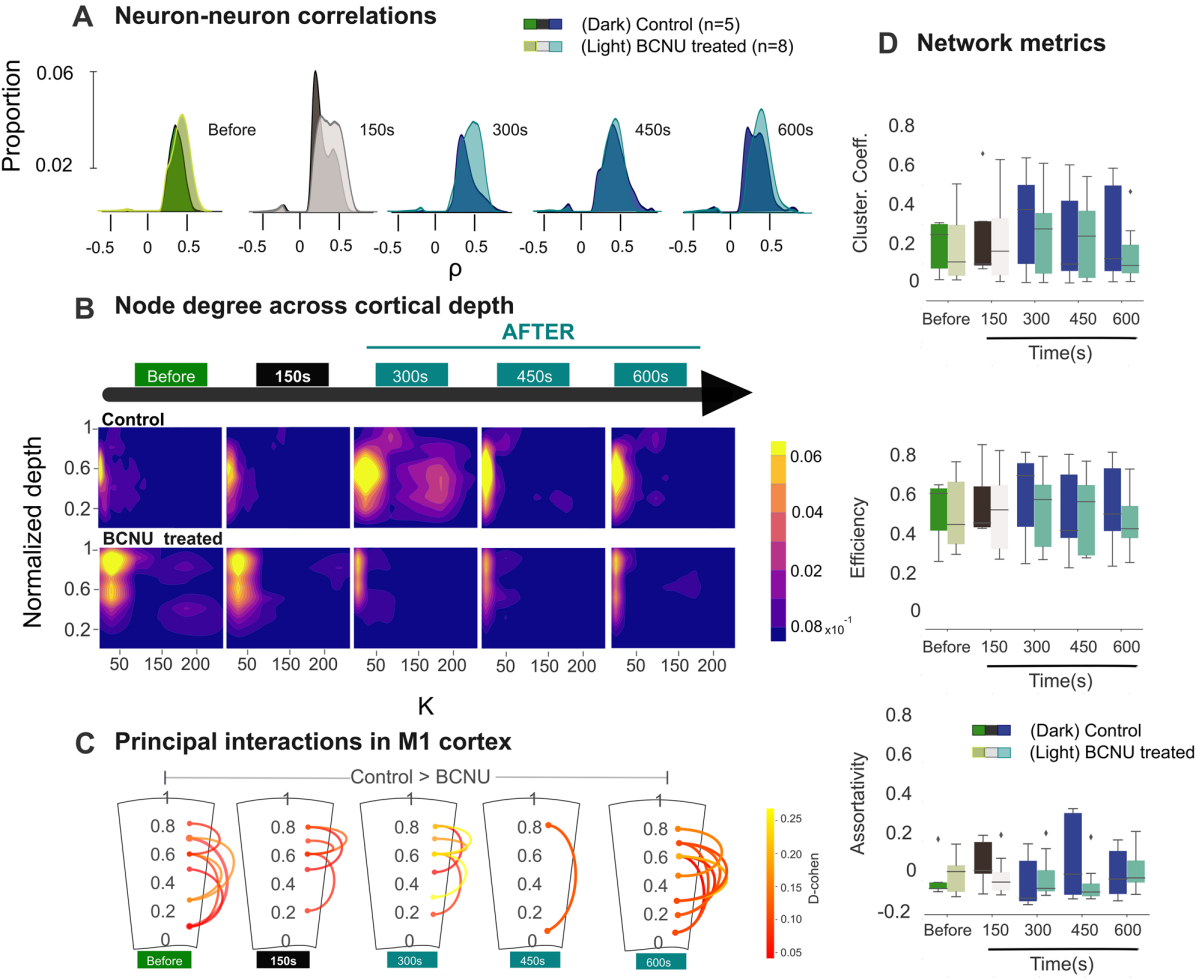
Neuron communication before, during, and after the hyperexcitable stimulus. (**A**) Distribution of group-specific neuron–neuron correlations (Spearman’s ⍴) across time windows (as defined in Fig. 3; pilocarpine stimulus within the second window). (**B**) Density plots of neuron-wise mean connectivity degree values (k) across the cortical depth and over time windows. (**C**) Schematic representation of the cortex; curved lines indicate statistically significant reductions of layer-to-layer connectivity in experimental animals (p < 0.05), color-coded according to Cohen’s d. (**D**) Network metrics over time; no statistically significant between-group differences were identified.

Cellular activity in M1 cortex was recorded using a stereo fluorescence microscope (Control 648 ± 184, BCNU 864 ± 149 cells; Fig. 4A). Figure 4B shows the population activity (each line represents one neuron) when stimulated with pilocarpine (red arrow) and an example trace of a calcium signal in one control and one BCNU-treated neuron. Figure 4C represents the time series segmentation used in the analysis of Fig. 6. We evaluated the neuron-specific signal amplitude and reactivity to the hyperexcitable stimulus as a function of depth (Fig. 5) and described the temporal evolution of cell-to-cell communication (Fig. 6).

The overall amplitude of the baseline [Ca^2+^]_i_ signals was homogeneous throughout the cortical depth in control animals and was mostly concentrated at 10 dB. In contrast, BCNU-treated animals showed a bimodal distribution of baseline signal amplitudes (Fig. 5A,B). Moreover, pilocarpine-induced hyperexcitation triggered high-amplitude signals in control animals (mostly in layers V and VI). BCNU-treated animals, on the other hand, consistently exhibited a bimodal amplitude distribution similar to that seen during baseline conditions and this feature persisted during the evolution of post-stimulus activity (Fig. 5 and Supplementary Fig. 2).

### Evaluation of the network configuration in response to hyperexcitation

Using temporal segmentation, the evolution of neuronal communication within M1 before and after the pilocarpine stimulus was evaluated in five non-overlapping temporal windows, each lasting 150 s. BCNU-treated animals showed slightly higher correlation coefficients than control animals during baseline conditions (Fig. 6A). The pilocarpine stimulus induced a bimodal distribution of correlation coefficients in control animals, with both groups showing an increase in the proportion of high correlation coefficients. Only BCNU-treated animals maintained high correlation coefficients following pilocarpine washout (300 s), after which both groups exhibited similar distributions of correlation coefficients (450 s, 600 s).

The relationship between cortical depth and the temporal evolution of connectivity degree was also assessed (Fig. 6B). During basal activity, BCNU-treated animals showed neurons with more connections (i.e., higher node degree, k) than control animals, particularly in superficial cortical layers. Slight changes in node degree were detected immediately after the pilocarpine stimulus, which decreased the number of connections in both groups. At 300 s, the control networks dramatically increased their k-degree and the spread of those connections, representing the effect of the external stimulus. Finally, in the last window, the distribution of the positions and k-degree resembles the initial shape of the window before stimulus. In contrast, BCNU-treated animals showed a progressive decrease in node degree at all depths after the pilocarpine stimulus.

The apparent discrepancy between the increased correlation coefficients in BCNU-treated animals and their reduced node degree led us to evaluate the inter-layer connectivity and its temporal evolution. For that, the statistically significant neuron–neuron interactions of the controls were compared with those of BCNU-treated animals, aggregated across depths (Fig. 6C). Control animals consistently showed a higher node degree between depths than BCNU-treated animals. At baseline, control animals displayed more connections between cortical depths, which was mimicked in the last temporal window. The largest between-group differences in the number of connections across depth positions occurred 300 ms after the pilocarpine stimulus, reflecting a delayed response to hyperexcitation, reverting to the initial conditions in the last window. Finally, three network metrics that evaluate network properties were explored, namely clustering, connectivity efficiency, and assortativity (Fig. 6D). The clustering coefficient was lower in BCNU-treated rats in the last time window. Assortativity showed values that changed over time, with BCNU-treated rats displaying increased assortativity at baseline, followed by reductions after pilocarpine stimulation, although no significant differences were found between groups at this level.

## Discussion

We investigated the link between aberrant cortical architecture, neuronal morphology, and intra-cortical communication in a model of abnormal cortical development that resembles human FCD. Evaluation of the primary motor cortex at early life (P30) confirmed previously reported dyslamination and altered neuronal morphologies (i.e., slightly larger and rounder neurons; Fig. 1) and morphological signs of glial activation (Fig. 2), all relevant characteristics of BCNU-treated animals^7,16^. Moreover, cortical layer-specific antibodies not only revealed dyslamination but also the presence of neuronal clusters (Fig. 3). Analysis of neuronal population dynamics through calcium imaging demonstrated that the micro-anatomical alterations were accompanied by abnormal neuronal activity (Fig. 5) during basal activity and pilocarpine, and also during post-stimulus time evolution (Supplementary Fig. 2) and intra-cortical network interactions and dynamics (Fig. 6).

Despite having histopathological hallmarks that resemble human FCD type IIa, BCNU-treated animals do not develop spontaneous seizures^7,8,10–12,16^. However, they exhibit epileptiform activity in the hippocampus and neocortex late in brain development^8^, as well as evoked epileptiform discharges after (Gamma-aminobutiric-acid) GABA_A_ receptor blockade^12^. The lack of spontaneous seizures in the absence of a precipitating agent is common in other rodent models of cortical malformations^6,17–20^.

In line with previous findings, none of our animals triggered spontaneous seizures. However, they displayed altered cortical activity, as evidenced by [Ca^2+^]_i_ imaging. At the individual level, cells in the control cortexes showed a different amplitude distribution at baseline, with a subset of neurons modifying their amplitude after the pilocarpine stimulus. Furthermore, cells recorded from BCNU-treated animals showed a bimodal distribution during baseline activity and also during the external stimulus. This could be explained by aberrant intracortical circuits due to cortical malformations, but it is also possible that BCNU directly altered the Ca^2+^ metabolism of neurons (see below). Nonetheless, the presence of altered activity (observed here via calcium activity and in previous reports via direct electrophysiological recordings^8^ without seizures) is reminiscent of the human condition, in which this congenital cortical injury often does not translate into clinical seizures until years after birth. Continued plasticity during postnatal cortical development likely unmasks altered neuronal activity and allows the propagation of epileptogenic discharges^21^.

Cortical dysplasias arise from altered processes related to cell migration and proliferation^22,23^. These alterations could result in abnormal patterns of intracortical communication and contribute to the development of epilepsy. The BCNU animal model has an important impact on one of these processes since induction occurs during the onset of cell migration. To approach the ways in which cellular functionality and communication could be altered under these conditions, in this work we employed large field-of-view calcium imaging, which helped us analyze the population dynamics and infer the connectivity of the entire depth of a specific neocortical region. Our results indicate that abnormal morphology and laminar organization entail repercussions for intra-cortical communication (Fig. 6C) in addition to alterations in individual neurons. This is evident in the anomalous connectivity patterns between neurons in BCNU-treated animals, which present differences in the numbers and strength of connections as well as the spread of connectivity (Fig. 6A,B). Such differences were present even before the stimulus and, moreover, were differentially modulated between groups by pilocarpine, with different patterns of return to baseline activity. The largest between-group differences were observed in the 300-s post-pilocarpine stimulation window, a result already expected from the knowledge that pilocarpine acts by a metabotropic response that involves binding to M_1_ muscarinic receptors, coupled to Gα proteins and that leads to a non-immediate response such as electrical stimulation and transmission; with control animals showing a transient increase in widespread connectivity (i.e., increased k-degree; Fig. 6B), whereas the number of connections did not increase in BCNU-treated animals. However, the connections seen in control animals during this timeframe, despite being increased in number, were less tightly coupled than those seen in BCNU-treated animals (i.e., lower correlation coefficients in control animals; Fig. 6A). While pilocarpine induces a spread of excitation throughout the cortex in control animals, the already altered intracortical circuits of BCNU-treated animals (as seen in the baseline condition) modulate the response to the pharmacological stimulation and do not permit the structure of intracortical connectivity to return to its original state. Global network metrics, however, did not differ between groups (Fig. 6D), likely due to the large size of the networks and the variability among specimens.

Throughout this work, we analyzed the time course of the [Ca^2+^]_i_ activity for each neuron, based on the assumption that alterations in neuronal Ca^2+^ homeostasis contribute to the pathogenesis of epilepsy because Ca^2+^ is both a messenger and signal transducer^24–27^. There is evidence that alteration of the intra-neuronal concentration of this ion during an external stimulus disrupts cell activity and impacts the activation of other membrane receptors^24^. These factors likely influence the temporal evolution of [Ca^2+^]_i_ signals and argue in favor of the analysis of time series rather than their deconvolution to derive neuronal events. In addition, direct between-group comparison of the [Ca^2+^]_i_ time series before, during, and after the pilocarpine stimulus allowed us to investigate the presence of pre-existing signaling alterations in BCNU-treated animals, as well as their response to a stimulus that led to hyperexcitability and the (lack of) return to baseline activity.

Our results are possibly related to BCNU-induced abnormal development, which can go beyond the organization of the cortex and alter several cellular pathways. Of note is the mTOR pathway, involved in the activation of calcium ATPases, like sarcoendoplasmatic Ca^2+^ ATPase (SERCA), plasma membrane Ca^2+^ ATPase and inositol 1,4,5-trisphosphate receptor type I, in neurons^28–30^. Recent advancements in the study of FCDs have confirmed the involvement of the mTOR pathway, which regulates physiological cellular functions such as cell growth, proliferation, ion channel expression, and synaptic and circuit plasticity^31–35^. Indeed, in utero electroporation for the introduction of the mutant MTOR transcript induces the generation of FCD and spontaneous seizures^36^, and the associated morphological alterations vary as a function of mTOR hyperactivation^37^. Notably, disruption of cortical layering is not a requisite for seizure generation, whereas the presence of dysmorphic neurons is^35^.

The BCNU animal model provides an analog of the microarchitectural disarrangement of human FCDs, but it is insufficient for a complete description of the epileptogenesis of such lesions. Nevertheless, we confirmed that altered microscopic anatomy of the cortex translates into altered neuronal activity and response to a pharmacological manipulation that leads to hyperexcitability in the primary motor cortex. Further work should evaluate other neocortical regions and the hippocampus, where previous reports have demonstrated anatomical alterations^7,9,11,16^. While the sample size investigated here is relatively small, the morphological features identified in M1 at P30 are in line with previous reports^7,16^. Moreover, recent work from our group in a larger n of BCNU-treated animals showed similar alterations of neuronal layering in the cortex, as well as alterations of myeloarchitecture^38^. A limitation of our work is the lack of characterization of the cells in our calcium imaging recordings. The identification of their excitatory or inhibitory nature, expression of markers of dysplastic neurons, as well as the changes in distribution and density of M_1_ in the cortex evaluated and morphological characteristics, and morphological characteristics, simultaneous with their activity over time, would enrich network analyses. This, however, represented a major technical challenge that we were not able to address.

In this study, we focused only on pilocarpine as a mechanism to perturb cortical activity, although there are other approaches to simulating seizure activity and hyperexcitability to understand epileptogenesis. Alteration of neuronal dynamics in live cortical slices can only be achieved with high concentrations of pilocarpine^39^, such as the one we used, thereby limiting the generalizability of our findings. Other pharmacological interventions should be explored to validate our results^40^. Recent animal models that induce FCD through in utero electroporation^41^, which produce dysmorphic neurons that can elicit and sustain seizure-like activity, should provide further insight into the generation and spread of abnormal activity within the cortex and allow for a better understanding of the anatomy and function of intracortical networks.

## Conclusions

Our work shows that in the BCNU animal model of cortical dysplasia, subtle morphological alterations at early life are linked to abnormal baseline neuronal activity and intracortical communication that, in turn, translate into aberrant responses to an external stimulus that causes hyperexcitation. Assessment of intracortical connectivity highlights the role of network dysfunction in the pathophysiology of cortical dysplasia. This contribution may guide the refinement of computational models of neocortical epileptogenesis and further improve our understanding of this common cause of focal epilepsy.

## Methods

### Animals

Protocols followed in this study were approved by the ethics committee of the Institute of Neurobiology, UNAM (Protocol #111-A) and carried out in accordance with federal regulatory laws for animal experimentation (NOM-062-ZOO-1999). All methods were compliant with the ARRIVE guidelines and regulations. Pregnant Sprague–Dawley rats were intraperitoneally injected with BCNU, n = 5 (Sigma-Aldrich; 20 mg/kg diluted in 5% sucrose) on gestational day E15. A single administration of this alkylating agent during the cortical development of the embryo results in cortical malformations^7^. Control animals (n = 3) were injected with corresponding volumes of saline solution on the same gestational day. Litters resulting from rats injected with BCNU had similar mortality rates as controls, and the pups appeared and behaved normally, except for their slightly smaller size and lower body weight. Pups remained with their dam until weaning day (P21). All animals were housed in a room with a 12-h light/dark cycle and had free access to food and water. A total of 19 rats (Control: 6 males, 2 females; BCNU: 8 males, 3 females) were evaluated at P30. There were two forms of evaluation, each rat serving in only one, with sample sizes indicated in each corresponding section.

### Immunostaining

Rats (Control = 3, BCNU = 3) were perfused intracardially with 4% paraformaldehyde (PFA; Sigma-Aldrich). Then, the brains were removed and placed in a fresh 4% solution overnight. Next, they were transferred to a 20% sucrose (w/v) solution in PBS 1x (w/v) for 24 h, followed by a 30% sucrose (w/v) solution with the same duration. Tissues were frozen using absolute ethanol and liquid nitrogen and stored at − 20 °C until sectioning. Coronal slices (20 μm thick) were obtained with a cryostat microtome (Leica) near Bregma (9.20 mm). Sections were preserved in PBS 1x (w/v), then washed (5 × 10 min) with PBS 1x + Tween 20, 0.3% (w/v) (Sigma-Aldrich). Blocking was performed with PBS 1x, Triton X-100, and 0.3% (v/v) BSA 2% (blocking solution) for 45 min at room temperature, then incubated overnight at 4 °C with primary layer-specific antibodies (NBP1-86671, Foxp2; polyclonal 1:2000, NBP1-84004, Necab; polyclonal 1:500, Novus Biologicals) and ab104224 NeuN 1:350 (Abcam) and G3893, GFAP 1:350 (Sigma-Aldrich). Fluorescently tagged secondary antibodies (Molecular Probes, AlexaFluor, goat anti-rabbit -488, -555, and goat anti-mouse -647, diluted at 1:500 in blocking solution) were incubated for 4 h at 4 °C and cover-slipped with microscope cover glass. Double or triple immunofluorescence was assessed with a laser confocal microscope (Zeiss LSM 780 DUO).

### Calcium imaging

Rats (Control = 5, BCNU = 8) were deeply anesthetized with isoflurane and then decapitated. The brain was quickly removed from the skull and immediately placed into an ice-cold oxygenated (95% O_2_; 5% CO_2_) solution (in mM: 2.5 KCl, 28 NaHCO_3_, 1.25 NaHPO_4_, 7 MgCl_2_, 0.5 CaCl_2_, 235 sucrose). Using a vibratome, coronal slices (230 μm) of the medial motor cortex of each brain were obtained and kept at room temperature, continuously bathed with artificial cerebrospinal fluid (ACSF, pH 7.4) gassed with 5% CO_2_ and 95% O_2_ for 20 min. Then, the slices were incubated with the cell-permeable fluorescent Ca^2+^ indicator Fluo-4 AM (Molecular Probes/Invitrogen) at a final concentration of 14 μM (prepared from a 2 mM stock solution in dimethyl sulfoxide with 0.6% Pluronic F-127; Sigma-Aldrich). Dye loading proceeded in the incubator chamber at 37 °C and normal atmospheric conditions (95% O_2_; 5% CO_2_) for 1 h. Then, each slice was placed in the bottom of a Plexiglass chamber attached to the stage of the microscope and continuously perfused (2 ml/min) with gassed physiological solution ACSF 1x (in mM: 126 NaCl, 5 KCl, 2 CaCl, 2 MgSO_4_, 26 NaHCO_3_, 1.25 NaH2PO_4_, 10 glucose) at room temperature. We recorded the calcium activity with a stereoscopic fluorescence microscope (Leica M205 FCA) with a 2.0 × PlanApo objective (max. aperture 0.35), coupled to a CCD camera sampling at 3.3 Hz.

Time series were recorded at basal activity and during external stimulation in one slice per animal. We selected the 300 μM pilocarpine concentration based on a previously reported extracellular activity dose–response curve^39^. Each slice was recorded following the next sequence: (1) basal activity with ACSF perfusion (3 min); (2) a pilocarpine stimulus (30 s) followed by 13 min of ACSF perfusion; (3) a 15 min pause with ACSF solution without light stimulation; and (4) a KCl stimulus (30 s) and the rest of ACSF perfusion to assess the total sample of cells that had activity during the recordings. Only neurons showing activity during all stages of recording and responsive to the KCl stimulus were analyzed.

### Data analysis

#### Morphological evaluation

Using the anti-NeuN immunofluorescence label, we manually delineated the perimeter of each cell within a fixed-width region (500 μm) in M1 and stored it as a region of interest (ROI) with FIJI software^41,42^. We measured the depth of the center of mass of each ROI, which was normalized with respect to the thickness of the cortex for each specimen. To assess morphological changes commonly observed in dysplastic neurons, the area and roundness of each cell was obtained by fitting an ellipse to each ROI using FIJI^42^. Between-group differences for both shape descriptors were assessed using Student’s t-test on the pooled data comprising all neurons from all specimens, assessed at two different cortical depths (layers II–III and layer V, zones I and II).

#### Calcium activity and signal analysis

Neurons were manually selected by drawing ROIs, and the average pixel intensity (i.e., fluorescence) was extracted at each frame using FIJI^41,42^. Fluorescence values were normalized as ΔF/Fmin in each ROI, then a third-order Bessel low-pass filter was applied by Signals Scipy^43^ and corrected for bleaching of the signal through a linear regression.

The full waveform of the calcium time series was evaluated; for each, the frequency and power spectra were obtained using Welch’s method, and those values were correlated with the depth of each identified neuron, which was calculated using their corresponding ROI coordinates and normalized to the cortical thickness (0–1 values, where zero corresponds to the interface with white matter and one corresponds to the pial surface). In this evaluation, we separated the calcium time series into two major periods based on the time of stimulation. One was related to baseline and the other to the response after stimulation (i.e., after the pilocarpine stimulus). We assessed the statistical significance of each activity (basal and pilocarpine) between groups using Student's t-test. The post-pilocarpine period was subdivided into 150 s windows to show the evolution of changes (see Supplementary Fig. 2).

#### Time series selection, connectivity, and network evolution

Calcium time series were selected if their activity during the 30 s pilocarpine stimulus was considerably higher than baseline activity (two standard deviations from the maximum power value). Spectral analyses were conducted for each neuron’s time series. Correlations (Spearman’s ⍴) were used to infer cell-to-cell connectivity, and the statistical significance of these correlations was assessed by comparing them to an empirically created null distribution of correlations between synthetic signals (n = 1000 for each signal) constructed by the amplitude and phase values sampled from the real data; correlations outside two standard deviations from the null distribution mean were selected. The false discovery rate^44^ was used to further refine the resulting adjacency matrices by controlling for multiple comparisons. A connection was defined as a significant pairwise correlation after such correction, and the node degree (k) corresponds to the number of such connections.

The evolution of the resulting network was evaluated over five discrete time intervals: 0–150 s (before), 151–300 s (window 1), 301–450 s (window 2), 451–600 s (window 3), and 600–750 s (window 4). The first interval represents the time period without an external stimulus; the second one corresponds to the moment reflecting the acute effects of pilocarpine; and the last three intervals represent the rest of the time series. From these time-dependent adjacency matrices, we derived network metrics, namely efficiency of communication, cluster coefficient, and assortativity values, using NetworkX^45^ and R version 4.2.2^46^. Linear mixed effects, as implemented in lme4^47^, were used to evaluate the relationship between network metrics, the experimental group, and time. Group and time were entered into the model as fixed effects, while subjects were entered as random effects. Statistical significance was assessed with p values obtained by likelihood ratio tests of the full model with the effect in question versus the model without the effect in question.

#### Node degree evaluation and principal interactions

A depth-dependent analysis of the number of connections (node degree) per neuron was performed. For each time window, the normalized cortical thickness was divided into 0.1 steps, for which the average node degree was calculated relative to basal activity. Statistically significant differences were assessed using Student’s t-tests, and effect sizes were estimated with Cohen’s d^47,48^.

## Supplementary Information

Supplementary Figures.

## Data Availability

Calcium time series for all experimental animals and conditions are available at 10.5281/zenodo.7675887.
