# Supplementary material for: Altered functional connectivity and network excitability in a model of cortical dysplasia
Source: Sci Rep. 2023 Jul 30;13:12335. doi: 10.1038/s41598-023-38717-2 (PMC10387479; doi:10.1038/s41598-023-38717-2)
Supplement: Supplementary file 1 — Supplementary Figures. [file 41598_2023_38717_MOESM1_ESM.pdf]

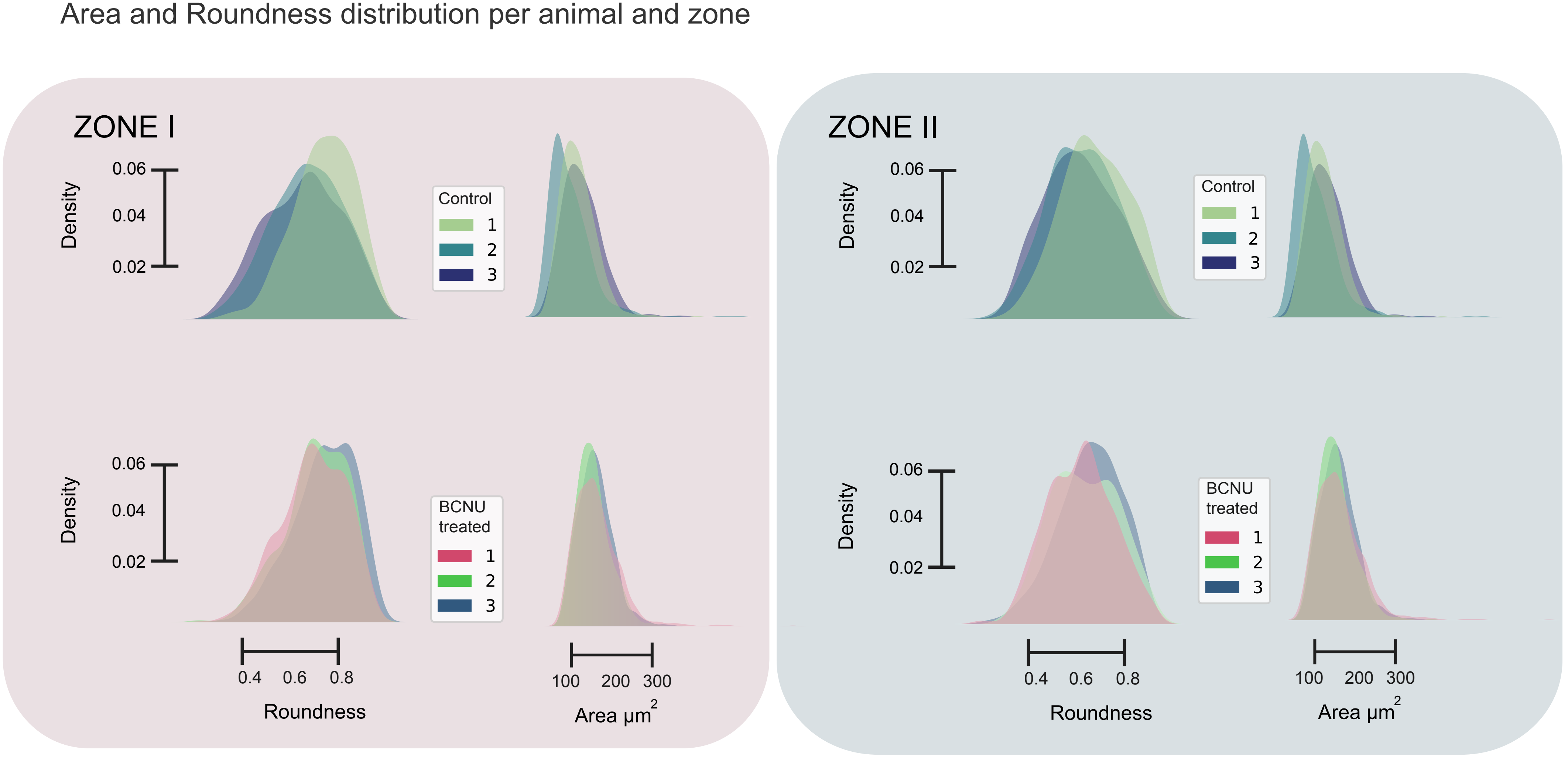

Supplementary Figure 1. Area and roundness distribution values per animal evaluated. The left panel sho
roundness distribution values and area distribution values in Zone I (as delimited in Fig. 1) for both groups (Control: left,

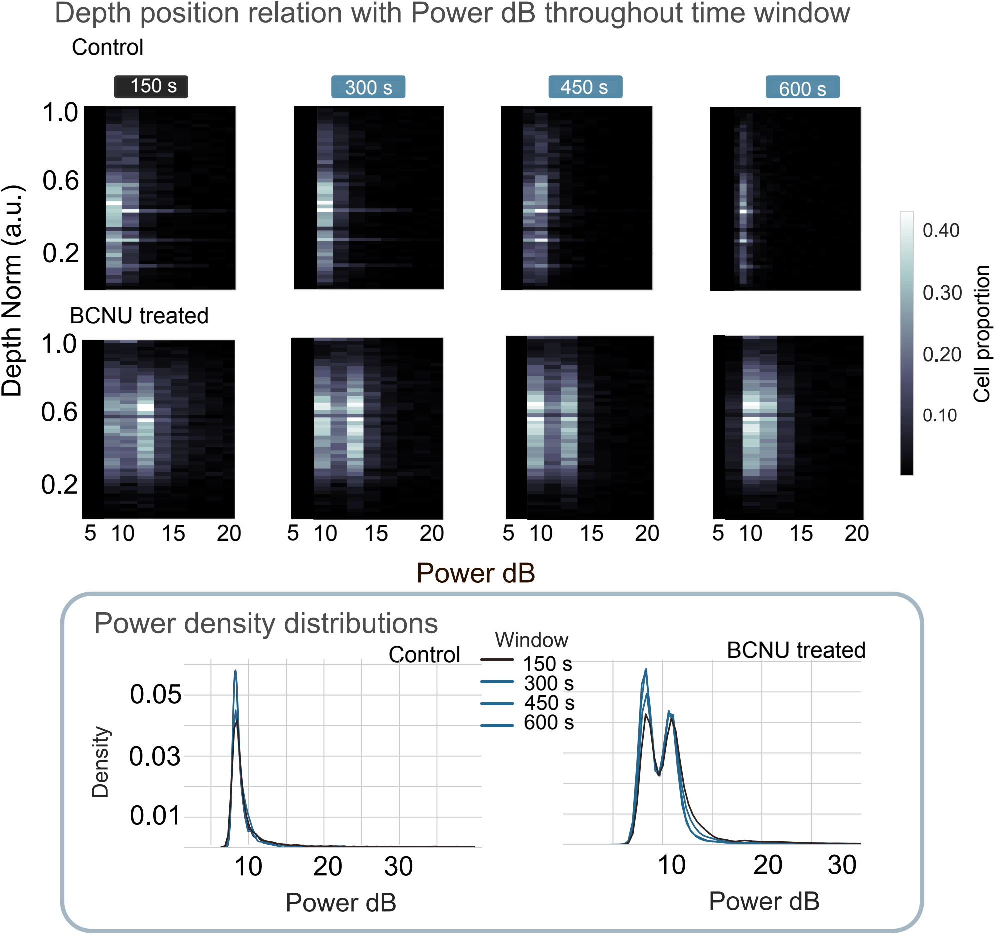

Supplementary Figure 2. Power values in activity post-pilocarpine stimulation wi[illegible] temporal evolution. The top panel shows the comparison between power values related to their depth position as shown in Figure 4, but with the temporal evaluation made in Figure 5. Temporal windows start with the pilocarpine stimulation (150 s), and are followed by the next 300 s, 450 s, and 600 s windows. The bottom section shows the cumulative distribution of power values colored according to the window time representation.
